# Quantitative trait loci associated with androgenic responsiveness in triticale (×*Triticosecale* Wittm.) anther culture

**DOI:** 10.1007/s00299-012-1320-2

**Published:** 2012-08-04

**Authors:** M. Krzewska, I. Czyczyło-Mysza, E. Dubas, G. Gołębiowska-Pikania, E. Golemiec, S. Stojałowski, M. Chrupek, I. Żur

**Affiliations:** 1 Institute of Plant Physiology, Polish Academy of Sciences, ul. Niezapominajek 21, 30-239 Kraków, Poland; 2 Department of Genetics and Cytology, Institute of Biology, Pedagogical University, Kraków, Poland; 3 Department of Plant Genetics, Breeding and Biotechnology, West Pomeranian University of Technology, Szczecin, Poland; 4 Department of Genetics, Faculty of Biotechnology, University of Rzeszow, Rzeszow, Poland

**Keywords:** Androgenesis responsiveness, Composite interval mapping, QTL, Marker-assisted selection, Winter triticale

## Abstract

Quantitative trait loci (QTLs) associated with androgenic responsiveness in triticale were analyzed using a population of 90 DH lines derived from the F1 cross between inbred line ‘Saka 3006’ and cv. ‘Modus’, which was used in a number of earlier studies on molecular mapping in this crop. Using Windows QTL Cartographer and MapQTL 5.0, composite interval mapping (CIM) and association studies (Kruskal–Wallis test; K–W) for five androgenesis parameters (androgenic embryo induction, total regeneration and green plant regeneration ability, and two characteristics describing final androgenesis efficiency) were conducted. For the studied components of androgenic response, CIM detected in total 28 QTLs which were localized on 5 chromosomes from A and R genomes. Effects of all QTLs that were identified at 2.0 or above of the LOD score explained 5.1–21.7 % of the phenotypic variation. Androgenesis induction was associated with seven QTLs (LOD between 2.0 and 5.8) detected on chromosomes 5A, 4R, 5R and 7R, all of them confirmed by K–W test as regions containing the markers significantly linked to the studied trait. What is more, K–W test revealed additional markers on chromosomes: 5A, 2BL, 7B and 5R. Both total and green regeneration ability were controlled by genes localized on chromosome 4A. Some of the QTLs that affected final androgenesis efficiency were identical with those associated with androgenic embryo induction efficiency, suggesting that the observed correlation may be either due to tight linkage or to pleiotropy.

*Key message* Five regions of the triticale genome were indicated as revealing significant marker/trait association. Markers located in these regions are potentially useful for triticale breeding through marker-assisted selection.

## Introduction

One of the features that distinguishes plants from animals is the fact that the majority of differentiated plant cells do not lose their developmental potentialities and under specific conditions can return to a totipotent embryogenic state. One of such processes called ‘microspore embryogenesis’/‘androgenesis’ starts with the reprogramming of male gametophytic cells (microspores). As a result, instead of mature pollen grains, the microspore forms an androgenic structure (AS) that closely resembles a zygotic embryo or can sometimes be similar to callus tissue. In both cases ASs can regenerate into haploid (*n*) plants and then after genome doubling—into doubled haploids (DH, 2*n*). Very fast—within one generation—production of totally homozygous lines makes the DH technology a very valuable tool for breeding practise, as well as for biotechnology and molecular studies.

In the last decade, many aspects of the androgenic process have been studied widely to bring the better understanding of the mechanisms that induce the switch from gametophytic to sporophytic developmental pathway. However, our knowledge of the nature of this process is still rather scant and random. Although DH populations are commonly used as models in gene mapping and genetic map construction, the identification of genetic factors controlling androgenic responsiveness and effective production of DHs is still not finished. The very strong dependence on the donor plant genotype suggests that the trait is controlled by a complex of genes with quantitative inheritance. In the case of many genotypes, sometimes of great breeding importance, this fact significantly limits the applicability of DH technology on a large scale. Identification of quantitative trait loci (QTLs) through interval mapping technology can indicate genome regions associated with androgenic responsiveness. Determination of markers linked to this trait and estimation of their phenotypic effects could bring significant progress to DHs utilization. Identification of molecular markers linked with high effectiveness of the process can be used to determine the value of a genotype in a breeding program and in marker-assisted breeding aimed at transferring genes from highly responsive genotypes to the recalcitrant ones (Bolibok and Rakoczy-Trojanowska 2006). It is also possible to apply the selected markers in physical mapping, map-based cloning and isolation of the gene of interest.

Early studies on the genetic control of androgenesis suggested the involvement of several independent nuclear genes with additive or dominance effects which interacted with environmental factors and could be modified by cytoplasmic background (Foroughi-Wehr et al. 1982; Agache et al. 1989; Powell 1988, 1990; Larsen et al. 1991; Hou et al. 1994; Ferrie et al. 1995). It has become evident that the final efficiency of the androgenesis process depends on three independent components: the efficiency of AS formation, AS regeneration ability and the frequency of green plant regeneration. Each of them stays under separate genetic control (Henry et al. 1994). It has been supposed that the control of AS formation shows mainly nuclear inheritance, while the regeneration process is under strong environmental influence (Ryőppy 1996). Many studies have been focused on identification of parameters important for effective induction of androgenesis and plant regeneration but in most of them, the optimization of the protocol remained mostly empirical without going into the details of underlying mechanisms.

Triticale (×*Triticosecale* Wittm.) is an intergeneric hybrid derived from a cross between wheat (*Triticum* e.g. *T. turgidum*) and rye (*Secale cereale*). It has been developed into a commercially valuable feed grain crop due to a combination of several advantageous features: high yield potential, good grain quality and relatively high tolerance to unfavorable environmental conditions. As a consequence of still growing economical importance of triticale, numerous research papers focused on its genetics and genome organization have been published in the last decade (Góral et al. 2005; Tams et al. 2005; Alheit et al. 2011; Badea et al. 2011; Tyrka et al. 2011). However, according to our knowledge, there is only one report concerning the mapping of QTLs for androgenic response in this cereal (González et al. 2005).

In the presented experiment, ‘Saka 3006’×’Modus’ triticale mapping population of 90 DH lines of hexaploid winter triticale (×*Triticosecale* Wittm.) derived via wide crossing with maize was used as the model for anther culture response evaluation. Then, a recently produced well-saturated genetic linkage map (Tyrka et al. 2011) for the same triticale population was used to identify and locate QTLs associated with androgenic responsiveness.

## Materials and methods

### Donor plants and growth conditions

The triticale mapping population was created by the team of Dr. Eva Bauer from the State Plant Breeding Institute, Hohenheim University, Germany. Two hexaploid winter triticale: inbred line ‘Saka 3006’ and cv. ‘Modus’ were crossed and then several F1 plants were used to develop DH lines by the maize method (modified after Wędzony 2003). The same method was used to produce DH lines from both parental genotypes. For 90 DH lines ‘Saka 3006’ × ‘Modus’, genetic map was constructed which consists of 1,568 markers (155 SSRs, 28 AFLPs, 1,385 DArTs) distributed within 21 linkage groups. The map covers 2,397 cM and the average distance between markers is 4.1 cM (Tyrka et al. 2011).

Germinating triticale kernels after 2 days in the dark at room temperature were placed in perlite pre-soaked with Hoagland’s salt solution and vernalized for 7 weeks at 4 °C and 8/16 h (day/night) photoperiod. The seedlings were then planted into pots containing a mixture of soil, de-acidified substrate peat and sand (2/2/1; v/v/v) and grown in a greenhouse [temperature 20 ± 2 °C, 16/8 h (day/night) photoperiod] until the flowering. Additional illumination (Philips 400 W Son-T Agro High Pressure Sodium Lamp) was applied to prolong the light period or as supplementary light during unfavorable weather conditions.

The experiment was carried out in three independent replications in various vegetation seasons [tiller collection started (first) at the end of April 2009, (second) in mid-January 2010 and (third) in the first decade of October 2010] to evaluate the effect of uncontrolled endogenous and environmental factors.

### Androgenesis induction phase

Tillers were collected when the majority of microspores were at the mid- to the late-uninucleate stage of development. The tillers were then wrapped in plastic bags, placed in jars containing Hoagland’s salt solution and stored at 4 °C, in the dark for 3 weeks. Subsequently, the spikes were sterilized with 96 % ethanol. Aseptically excised anthers were placed in 60 × 15 mm Petri dishes (100 anthers from one spike per dish) with modified C17 induction medium (Wang and Chen 1983). The medium was supplemented with 0.5 mg/l kinetin, 1 mg/l Dicamba and 1 mg/l Picloram, 90 g/l maltose and 0.6 % agar; pH 5.8. The cultures were incubated in the dark at 28 ± 1 °C.

### Androgenesis regeneration phase

Androgenic structures (AS) of size >1 mm were transferred successively starting from the sixth week of cultivation at 3-week intervals. The structures were put into 90 × 20 mm Petri dishes (30 AS per a dish) with regeneration medium modified 190-2 (Zhuang and Xu 1983) containing 30 g/l sucrose, 0.5 mg/l kinetin, 0.5 mg/l NAA and 0.6 % agar, pH 6.0. Regeneration phase took place at 26 °C, in light (at about 30 μmol m^−2^ s^−1^ during the first week, then increased to 80–100 μmol m^−2^ s^−1^) with 16/8 h (day/night) photoperiod.

The parameters describing androgenesis responsiveness were evaluated for each DH line. The spikes (at least 6) were collected from 4 to 6 plants; from each spike 100 anthers were placed on medium and cultured.

### Statistical and QTL analysis

The effectiveness of androgenesis induction was based on the number of androgenic structures (AS) produced per 100 anthers of the donor plant (AS/100A). Each dish containing 100 anthers collected from one spike was assumed to be a replicate. For the majority of DH lines, mean values were calculated from six replicates for each experiment. The effectiveness of regeneration phase was expressed in the total number of regenerants and in the number of green regenerants per 100 androgenic structures transferred to regeneration medium (R/100AS; GR/100AS). The final androgenic responsiveness was expressed in the total number of regenerants and in the number of green regenerants per 100 isolated anthers (R/100A; GR/100A).

Statistical analysis of results was performed using Statistica version 8.0. For each parameter (AS/100A, R/100AS, GR/100AS, R/100A, GR/100A), the normal distribution of scores has been verified by Shapiro–Wilk test to validate the use of the parametric tests. The effect of tested variable/variables was examined by one-way or multi-factor analysis of variance (ANOVA). Post-hoc comparison was conducted with the use of Duncan’s multiple range test (*p* ≤ 0.05).

To express repeatability of the analysis (compare the degree of variation from one replication to another), the coefficient of variation (CV) for each DH line was calculated according to the formula: 100 × [(standard deviation of array)/(average of array)] = CV (%).

The relationship between segregations of single marker and trait was analyzed with the Kruskal–Wallis test (K–W) using MapQTL 5.0 package (Van Ooijen 2004). Linkage analysis was performed using the composite interval mapping (CIM) method with Windows QTL Cartographer version 2.5 (Wang et al. 2007). A LOD threshold of 3.0 was used to detect QTL; however, in the case of consistent detections a LOD score beyond 2.0 was considered significant. The percentage of phenotypic variation was calculated with a single factor regression (*R* ^2^). The CIM and Kruskal–Wallis test analyses were performed separately for each experimental replication.

## Results

### Androgenesis efficiency

The analysis of androgenic responsiveness among studied DH lines showed a great variation in all components of this feature: androgenic structure induction, total plant regeneration and green plant regeneration ability. Moreover, even though all three experiments were carried out under the same greenhouse conditions, ANOVAs showed highly significant differences (*p* < 0.001) between the replications.

The obtained results confirmed significant variation in androgenic responsiveness between parental genotypes and among their progeny. The average androgenesis induction efficiency calculated out of three replications for DH ‘Modus’ is almost three times higher in comparison with DH ’Saka 3006’ (110 and 40 AS/100A, respectively). Average total regeneration ability was similar for parental genotypes (15 and 11 R/100AS, respectively) but the number of regenerated green plants was almost two times higher in the case of DH line ‘Modus’ (8 vs. 4.5 GR/100AS). It resulted in almost three times higher final androgenesis efficiency for this genotype in comparison with DH line ‘Saka 3006’ both in respect of total and green regenerants production (12 vs. 4 R/100A and 2 vs. 6 GR/100A).

The DH progeny population presented a much wider spectrum of responses: the mean AS production per 100 anthers ranging from 8 to 169, and mean regeneration ability from 4 to 30 for R/100AS and from 1 to 18 for GR/100AS.

Table 1 summarizes the results obtained in each replication of the experiment for the parental genotypes and their offspring population. The distribution of almost all parameters used for androgenesis capacity evaluation was near to normal, skewed slightly towards the lower part of the frequency scale. However, as no procedure of data transformation resulted in a more normal distribution, the original data has been used for further analysis.

**Table 1:** Efficiency of androgenesis in parental triticale genotypes (DH ‘Saka 3006’, DH ‘Modus’) and derived from their F1 cross hybrid population of 90 DH lines received in three separate experiments

Trait	Exp.	DH Saka 3006	DH Modus	Offspring DH population			
		Mean ± SD		Mean ± SD	Range	Skewness	Kurtosis
AS/100A	1st	72.5 ± 28.3	25.3 ± 5.2	85.5 ± 5.8	0–224	0.43	−0.55
	2nd	13.2 ± 2.8	131.0 ± 8.2	59.7 ± 3.8	2–155	0.45	−0.35
	3rd	43.4 ± 14.3	116.6 ± 42.5	87.9 ± 6.4	2–251	0.60	−0.45
GR/100A	1st	2.1 ± 0.9	3.3 ± 3.3	4.5 ± 0.5	0–22	1.88	4.08
	2nd	1.0 ± 0.7	8.0 ± 1.6	4.9 ± 0.4	0–17	0.80	0.33
	3rd	2.0 ± 1.1	6.0 ± 3.4	5.2 ± 0.6	0–38	2.69	11.01
GR/100AS	1st	3.7 ± 1.4	13.9 ± 13.9	6.0 ± 0.5	0–19	0.75	−0.07
	2nd	4.3 ± 3.0	7.2 ± 1.4	9.8 ± 0.7	0–31	0.78	0.78
	3rd	5.6 ± 2.7	3.9 ± 1.2	6.5 ± 0.5	0–17	0.71	−2.85
R/100A	1st	4.7 ± 1.1	4.0 ± 4.0	8.4 ± 0.6	0–25	0.86	0.35
	2nd	2.0 ± 0.8	18.0 ± 2.2	9.2 ± 0.6	0–22	0.23	−0.85
	3rd	4.8 ± 1.4	14.7 ± 7.9	10.0 ± 0.8	0–46	1.56	4.15
R/100AS	1st	8.7 ± 2.6	16.7 ± 6.7	12.0 ± 0.7	0–34	0.66	1.34
	2nd	11.3 ± 4.2	16.2 ± 2.0	18.5 ± 0.9	0–47	0.41	1.33
	3rd	12.3 ± 3.5	11.0 ± 2.5	13.6 ± 0.7	0–35	0.74	1.17

Data for parental genotypes are the mean of six replicates ±SD. The offspring DH population is characterized by the mean value ± SD, the extremes range (min–max) and parameters of the data distribution (skewness and kurtosis)

*AS/100A* the number of androgenic structures (AS) produced per 100 anthers (A) of the parent plant, *R/100A* total number of regenerants (R) per 100 anthers (A), *R/100AS* total number of regenerants (R) per 100 androgenic structures (AS) transferred to the regeneration medium, *GR/100A* number of green regenerants (GR) per 100 anthers (A), *GR/100AS* number of green regenerants (GR) per 100 androgenic structures (AS) transferred to the regeneration medium

To compare the degree of variation from one replication to another, CV was calculated separately for each DH line and each androgenesis parameter. The results summarized in Table 2 suggest that both androgenic induction efficiency and total regeneration ability are characterized by similar CV distribution. About 30 % of DH lines reacted consistently in all three experimental replications, ca. 40 % were less stable; the results obtained for 15–25 % of the DH lines were rather inconsistent, but only 5–11 % of DH lines were highly influenced by uncontrolled factors. Among tested parameters, green plant regeneration ability (GR/100AS) was characterized by the highest variation across three experimental replications with almost 30 % of DH lines reacting in a very inconsistent manner.

**Table 2:** Distribution of 90 triticale DH and both parental lines according to the coefficient of variation (CV) for the parameters describing the effectiveness of androgenesis across three independent experimental replications

CV (%)	Number of DH lines		
	AS/100A	R/100AS	GR/100AS
0–25	28	27	17
25–50	36	41	31
50–75	23	14	19
>75	5	10	25

CV (%) = 100 × [(standard deviation of array)/(mean of array*)]

mean of array* the mean for each parameter and each DH line calculated from three independent experimental replications, *AS/100A* the number of androgenic structures (AS) produced per 100 anthers (A) of the parent plant, *R/100A* total number of regenerants (R) per 100 anthers (A), *R/100AS* total number of regenerants (R) per 100 androgenic structures (AS) transferred to the regeneration medium, *GR/100A* number of green regenerants (GR) per 100 anthers (A), *GR/100AS* number of green regenerants (GR) per 100 androgenic structures (AS) transferred to the regeneration medium

### Mapping of QTLs

**Fig. 1:**
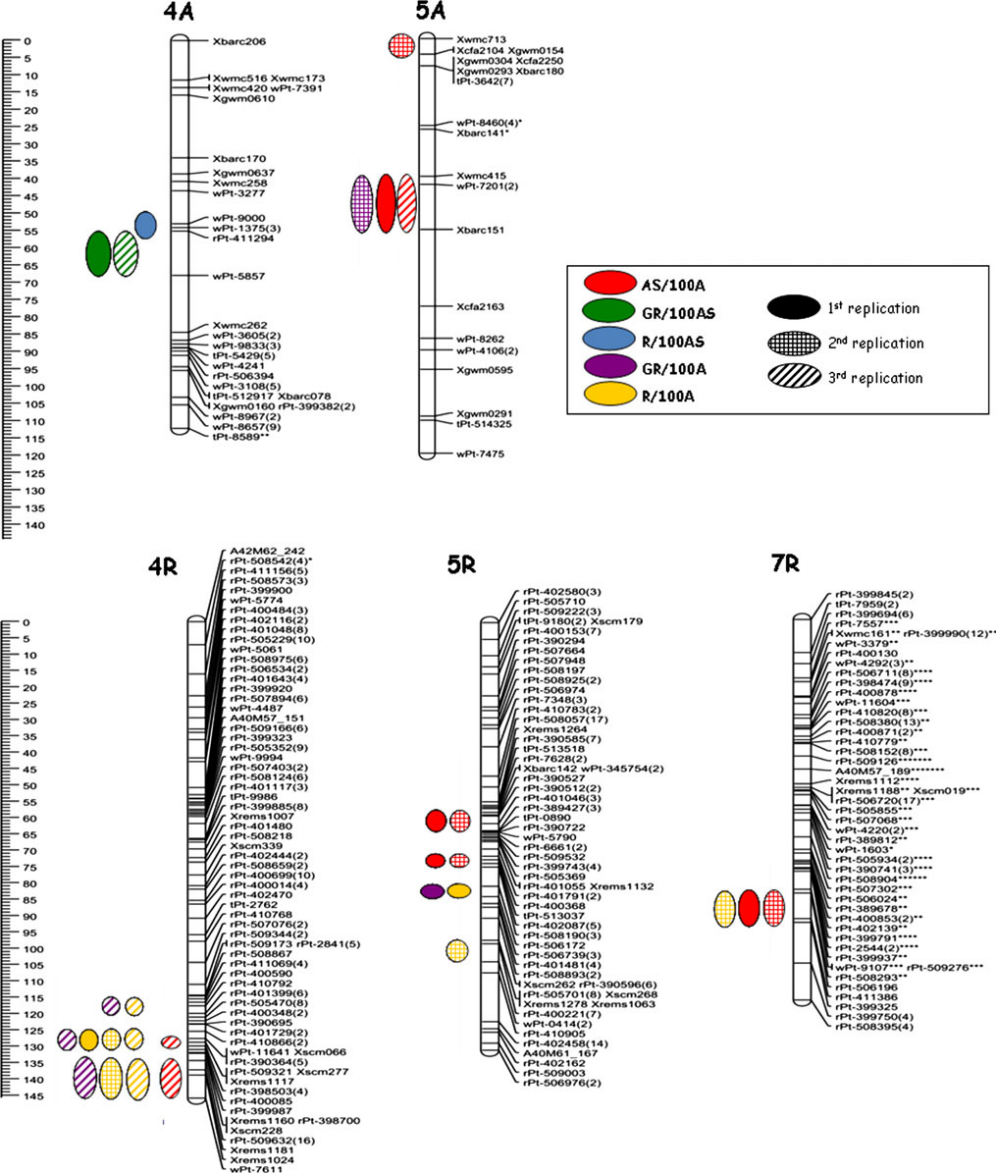
Chromosome maps showing locations of QTLs associated with androgenic responsiveness in anther culture of triticale (×*Triticosecale* Wittm.) Saka 3006/Modus mapping population. Results of Composite Interval Mapping for each studied traits: AS/100A (average number of androgenic structures per 100 anthers), GR/100AS (average number of green regenerants per 100 androgenic structures), R/100AS (average number of total regenerants per 100 androgenic structures), GR/100A (average number of green regenerants per 100 anthers), R/100A (average number of total regenerants per 100 anthers) in three replications. The *ovals filled with color* mean first replication, *checkered ovals* mean second replication and *ovals with diagonal lines* mean third replication of experiment (color version online)

Summarizing the results of CIM analysis from all three experimental replications, 28 QTLs were detected and localized on 5 chromosomes from A and R genomes for all studied components of androgenic response (Table 3; Fig. 1). Additionally, the analysis using Kruskal–Wallis test revealed some statistically significant markers (*p* < 0.005) located on chromosomes 2BL and 7B (Table 4).

**Table 3:** Main characteristics of significant QTLs for the studied traits in triticale mapping population of 90 DH lines Saka3006 × Modus and their parents

Trait	Exp.	QTL	Flanking markers (cM)^a^	LOD	The position (cM) of the max. LOD value	*R* ^2^ (%)	Add	Marker linked to a QTL			
								Marker name^b^	Significance in K–W test	Mean value of genotypes	
										Maternal	Paternal
AS/100A	1st	*QASsm-5A-1*	Xwmc415 (40)–Xbarc151 (55)	5.0	40	13.6	−21.10	Xwmc415	0.0001	58.7	106.1
		*QASsm-5R-1*	rPt-390527(58)–wPt-5790 (64)	5.8	59	16.2	22.30	rPt-390512	0.001	104.5	61.9
		*QASsm-5R-2*	rPt-400368(71)–rPt-508190 (75)	2.6	71	7.4	22.73	rPt-400368	0.0005	104.7	59.1
		*QASsm-7R-1*	rPt-508293 (82)–rPt-399325 (93)	2.0	83	5.1	−12.92	rPt-506196	0.05	71.4	102.8
	2nd	*QASsm-5A-2*	Xwmc713 (0)–Xgwm0154 (4)	3.8	0	10.7	−11.87	Xwmc713	0.0001	44.3	75.7
		*QASsm-5R-1*	rPt-390527 (58)–wPt-5790 (64)	3.0	64	8.9	10.86	wPt-5790	0.05	66.8	48.1
		*QASsm-5R-2*	rPt-400368 (71)–rPt-508190 (75)	4.3	74	12.4	12.65	rPt-508190	0.001	71.3	45.9
		*QASsm-7R-1*	rPt-508293 (82)–rPt-399325 (93)	4.5	82	12.8	−13.24	rPt-508293	0.0005	49.4	78.3
	3rd	*QASsm-5A-1*	Xwmc415 (40)–Xbarc151 (55)	4.5	42	13.1	−22.91	wPt-7201	0.001	63.7	108.6
		*QASsm-4R-1*	wPt-11641 (127)–rPt-398503 (130)	3.9	129	12.0	−21.27	rPt-398503	0.0005	61.7	111.3
		*QASsm-4R-1*	rPt-509632 (134)–wPt-7611 (146)	5.1	140	16.0	−24.63	Xrems1024	0.0001	69.9	116.3
R/100AS	1st	*QRASsm-4A-1*	wPt-9000 (50)–rPt-411294 (57)	4.4	53	15.7	3.36	wPt-9000	0.005	14.0	10.0
R/100A	1st	*QRsm-4R-1*	rPt-410866 (125)–rPt-398503 (130)	2.5	127	8.8	−1.85	rPt-390364	0.05	6.4	10.3
		*QRsm-5R-1*	rPt-506172 (80)–rPt-506739 (84)	2.4	83	8.3	1.77	rPt-506739	0.01	10.3	6.4
	2nd	*QRsm-4R-1*	rPt-410866 (125)–rPt-398503 (130)	2.9	128	8.0	−1.49	Xrems1117	0.05	7.7	10.6
		*QRsm-4R-2*	rPt-509632 (134)–wPt-7611 (146)	2.5	138	7.1	−1.41	Xrems1024	0.05	7.8	10.4
		*QRsm-5R-2*	rPt-505701 (97)–wPt-0414 (104)	4.5	97	13.3	1.98	rPt-505701	0.0005	10.9	7.0
		*QRsm-7R-1*	rPt-508293 (82)–rPt-399325 (93)	4.3	87	13.4	−1.98	rPt-506196	0.001	7.6	11.5
		*QRsm-4R-1*	rPt-410866 (125)–rPt-398503 (130)	5.6	128	18.1	−3.29	Xrems1117	0.0001	6.4	13.4
		*QRsm-4R-2*	rPt-509632 (134)–wPt-7611 (146)	5.3	134	17.2	−3.22	rPt-509632	0.0001	5.9	13.2
		*QRsm-4R-3*	rPt-411069 (115)–rPt-401399 (120)	6.2	117	19.7	−3.47	rPt-410792	0.0001	5.8	12.7
GR/100AS	1st	*QGRASsm-4A-2*	rPt-411294 (55)–wPt-5857 (69)	3.6	69	13.1	1.62	wPt-5857	0.005	7.2	4.3
	3rd	*QGRASsm-4A-2*	rPt-411294 (55)–wPt-5857 (69)	4.2	55	13.4	1.67	rPt-411294	0.05	7.7	5.0
GR/100A	1st	*QGRsm-5R-3*	rPt-506172 (80)–rPt-506739 (84)	4.1	83	13.1	1.67	rPt-506739	0.001	6.1	3.0
	2nd	*QGRsm-5A-1*	Xwmc415 (40)–Xbarc151 (55)	3.8	55	12.2	−1.43	Xbarc151	0.005	3.4	6.1
	3rd	*QGRsm-4R-1*	rPt-410866 (125)–rPt-398503 (130)	6.8	128	21.7	−2.84	Xrems1117	0.0005	2.8	7.3
		*QGRsm-4R-2*	rPt-509632 (134)–wPt-7611 (146)	6.3	134	20.4	−2.72	rPt-509632	0.0005	2.5	7.2
		*QGRsm-4R-3*	rPt-411069 (115)–rPt-401399 (120)	6.4	119	20.7	−2.74	rPt-401399	0.0005	2.8	7.0

^a^cM position of the marker on the genetic map of a given chromosome

^b^The closest marker to LOD peak

*LOD* logarithm of the odds for peaked marker, *R* ^*2*^ *(%)* % of phenotypic variance explained by the QTL, *Add* additive effect of the ‘Saka 3006’ allele

**Table 4:** Additional significant markers associated with studied traits revealed in Kruskal–Wallis test

Trait	Chromosome	Marker name	Position (cM)	*p* value	Mean value of maternal genotype (Saka)	Mean value of paternal genotype (Modus)
AS/100A	2BL	Xwmc658	0.000	0.005	72.4751	103.557
		wPt-8089	4.446	0.005	69.7389	103.146
	7B	wPt-6728	125.284	0.001	78.2233	50.3108
		wPt-4877	126.429	0.0005	113.450	66.7119
R/100A	5A	Xwmc713	0.000	0.001	7.34378	10.9818
		wPt-7201	42.281	0.0005	7.07415	10.8109
GR/100A	7B	wPt-1196	125.284	0.0005	8.31926	3.47577
	5R	tPt-513037	72.587	0.0005	6.03902	2.80556
		Xrems1063	97.154	0.0001	6.31125	3.04150
		rPt-400221	98.265	0.0005	6.16041	3.05026

The QTLs associated with the ability of androgenic structure production (AS/100A) were localized on four chromosomes: 5A, 4R, 5R and 7R. None of them was significant across all three replications (Table 3; Fig. 1). Quantitative trait locus QASsm-5A-1 located on the chromosome 5A was detected in the first and third experiments. It shows a positive effect when the allele comes from cv. ‘Modus’ and depending on the replication explained 13.1–13.6 % of the phenotypic variation. The LOD value varied between 4.5 and 5.0 and peaked at SSR marker Xwmc415 and at DArT marker wPt-7201. In the second experiment, another QTL for AS/100A was identified on the chromosome 5A. It was located in the telomeric region of the short arm of the chromosome close to marker Xwmc713. Two other QTLs for the studied parameter were identified in two out of three experimental replications on chromosomes 5R and 7R. Their effects explain up to 16.2 (5R) and 12.8 % (7R) of the variances but positive effect was inherited from different parents (Table 3). In the third replication of QTL analysis, two highly significant QTLs on chromosome 4R were identified with values of LOD 3.9 and 5.1, respectively. These QTLs explained 12–16 % of phenotypic variation and showed positive effect of alleles originated from the cv. ‘Modus’ (Table 3).

The analysis of Kruskal–Wallis test confirmed significance of all QTLs detected with the use of CIM method (Table 3) and revealed four additional significant markers for induction phase in at least two replicates at significance level *p* < 0.01 (Table 4). Two of them (Xwmc658, wPt-8089) were located on chromosome 2BL and the next two (wPt-6728, wPt-4877) on chromosome 7B.

Major QTLs for the regeneration efficiency were detected by CIM method on chromosome 4A (Table 3; Fig. 1). QTL associated with total regeneration ability (R/100AS) was detected only in the first experiment, but it had high LOD value (4.4) and *R* ^2^ score 15.7 % (Table 3). The next QTL controlling green plant regeneration ability (GR/100AS) was identified in two experiments and was located in almost the same region of the 4A chromosome. This QGRASsm-4A locus explained over 13 % of phenotypic variation. For both QTLs related to regeneration efficiency ‘Saka 3006’ alleles were associated with the favorable effect.

The last two parameters (GR/100A and R/100A) describe the final efficiency of the androgenesis process. Genomic regions associated with these traits were localized on four chromosomes 5A, 4R, 5R and 7R. The majority of them were located closely to the QTLs involved in androgenic structure production (Fig. 1).

In the region on chromosome 4R identified by CIM method as the location of several QTLs connected with the final efficiency of androgenesis, many significant markers were also identified by Kruskal–Wallis test (Table 3). The highest LOD value was 6.8 for GR/100A and 6.2 for R/100A and explained 21.7 and 19.7 % of the existing phenotypic variance, respectively (Table 3). In both cases, the alleles that increase the efficiency of androgenesis were contributed by ‘Modus’. The next QTLs were detected on chromosome 5R. One of these QTLs, which peaked at DArT marker rPt-506739, coincided for both studied parameters, but with lower LOD value for R/100A (Table 3). Interestingly, the QTL for GR/100A detected in the second replication was located on chromosome 5A in the same region which was identified as associated with androgenesis induction phase in two other experiments (Fig. 1). Moreover, Kruskal–Wallis test revealed six markers (*p* < 0.005) on chromosomes 5A, 7B and 5R significantly associated with final efficiency of androgenesis (Table 4). In the second experimental replication, an additional QTL for R/100A was identified which was localized on chromosome 7R and coincided with QASsm-7R-1 for AS/100A.

## Discussion

Recently, a lot of interest has been focused on the identification of genetic factors controlling androgenic responsiveness and effective production of DH lines. New, molecular methods developed in the last decade, which use marker genes associated with a specific trait, brought new possibilities of recognizing molecular and physiological background of androgenesis process. Identified molecular markers linked to androgenic response can be used in breeding programs to characterize the genotypes of interest and in marker-assisted transfer of important alleles to recalcitrant genotypes (Bolibok and Rakoczy-Trojanowska 2006).

It is well known that all three components (androgenic structure production, its regeneration ability and frequency of green plant regeneration) constituting final androgenesis effectiveness are polygenically controlled and independently inherited (Ekiz and Konzak 1994). Both additive, dominant and maternal genetic effects as well as interaction with environmental factors have been reported (Chaudhary et al. 2003; Torp et al. 2001). High complexity of genomic control makes molecular analysis of androgenesis very difficult. One possible way to overcome the problems is the utilization of QTL analysis, which makes it possible to link complex traits to specific chromosome regions and to estimate phenotypic effect of the indicated quantitative trait loci (Miles and Wayne 2008). It also gives the possibility to distinguish whether phenotypic variation is the result of a few loci with large effects or a sum of many loci with minor effects. Moreover, through QTL analysis various interactions, e.g., genotype × environment or epistatic interactions between QTLs can be detected.

Majority of studies aimed at the identification of QTLs in crop plants have focused on agronomically important yield-related traits and abiotic/biotic stress resistance (Börner et al. 2002; Collins et al. 2008; Bariana et al. 2010). Some papers only concentrated on QTL analysis for the in vitro tissue culture response traits (for review see Bolibok and Rakoczy-Trojanowska 2006). The identification of chromosomes or chromosomal regions involved in the different stages of androgenesis were examined, e.g., in wheat (Ben Amer et al. 1997; Torp et al. 2001, 2004) and rye (Grosse et al. 1996). Up to now, however, only one such study has been performed for triticale (González et al. 2005). Moreover, as the molecular marker linkage map constructed for DH population used in the study of González et al. (2005) contained only 356 AFLP, RAPD, RAMP and SSR markers with an average of 1 marker for 6.9 cM, the reported results should be verified and completed.

Identification of molecular markers linked with genes of interest is much more efficient when a reasonably dense genetic map for segregating the population is available. Such a map (Tyrka et al. 2011) has been recently constructed for the ‘Saka 3600’ × ‘Modus’ DH population of hexaploid triticale derived by distant crosses with maize according to the method described by Wędzony (2003). The main goal of its production was the localization of drought resistance genes (Hura et al. 2009). Next, the same population was used as the model for molecular analysis of QTLs controlling the resistance to pathogenic fungus *Microdochium nivale* (Szechyńska-Hebda et al. 2011). The constructed map provides a satisfactory framework for the search of chromosome regions involved in the control of quantitative traits. The fact that the mapping population was created without using androgenesis-based methods protects it against over-representation of alleles favoring good responsiveness of this process. Screening of the androgenic responsiveness revealed substantial variation between parental genotypes and across its offspring population in all components of this feature: androgenic structure induction, total plant regeneration and green plant regeneration ability. It altogether makes the constructed map a useful tool for determining QTLs involved in the androgenic response.

Almost normal frequency of distribution of the majority of tested traits allowed for the utilization of CIM method (Windows QTLCartographer 2.5). The problem of skewed distribution observed in some cases could be theoretically overcome by transformation of the data. However, as the transformations usually applied (ln(*x* + 1); arcsin√*x*) did not improve the normality of the analyzed data distribution, the non-parametric Kruskal–Wallis test (MapQTL 5.0) was used for association studies.

Experimental design (3 separate replications in the same glasshouse conditions but at various vegetation seasons) revealed the variation in androgenic responsiveness induced by some uncontrolled environmental (the length of the natural day light, light intensity, humidity) or endogenous (e.g., plant condition, biological clock) factors or their interaction effects. On the one hand, it seems to be inconvenient for molecular analysis but on the other it makes it possible to identify only the most significant QTLs associated with the studied traits. It also revealed the range of variation induced by other than genomic factors and helped identify highly influenced parameters. In this case, it was the green regeneration ability which confirmed earlier suggestions that the regeneration process is under strong environmental effect (Ryőppy 1996).

In the studied triticale population, the regions involved in androgenic embryo induction detected on chromosomes 5A, 5R and 7R seem to be the most important in androgenic responsiveness. QTLs responsible for AS production were identified on chromosome 5A in all three experimental replications. QTLs indicated on 5R and 7R chromosomes were significantly associated with this trait in two out of three replications. The last two QTLs associated with androgenic embryo induction were detected on the 4R chromosome in only one experimental replication. Almost all detected QTLs on 5A, 5R and 7R explained over 10 % of the phenotypic variance. Kruskal–Wallis test confirmed significance of all detected QTLs and additionally revealed other markers associated with this trait localized on two chromosomes of the B genome (2BL, 7B).

The obtained results are in partial agreement with earlier reports. Zhang and Li (1984) identified genes located on several chromosomes (on 2A and 2D with major effect, and 5A, 5B, 4A and 2B having minor effect) that are involved in wheat embryo production. Lazar et al. (1987) demonstrated that factors enhancing in vitro response of immature embryos are located on 6R and 7R chromosomes and that the disomic additions of rye chromosome 4R into wheat genome enhanced the haploid embryo development. Regions on chromosomes 6R and 5R were also identified by Bolibok et al. (2007) as associated with the response of immature rye embryos. QTLs for induction phase and final efficiency of androgenesis located on chromosome 4R were detected also by González et al. (2005). Moreover, this chromosome seems to be connected with callus induction and somatic embryogenesis ability in rye tissue cultures (Bolibok et al. 2007). Börner et al. (1998) reported that chromosome 4R also includes several genes involved in the restoration of male fertility (*rfg1*) whereas other authors (Koebner and Martin 1989; Benito et al. 1994) reported identification of loci coding aminopeptidases playing a role in nutrient catabolism, metabolic activity that is important in early embryo development.

Different results were obtained by Agache et al. (1989) using monosomic chromosome substitution and translocation lines of wheat: androgenic embryo formation frequency was enhanced by genes on chromosome 1D and 5BL chromosome arm.

Our research shows that QTLs with a major effect on both total and green regeneration abilities are carried only on chromosome 4A. Relatively small number of detected associations can be explained by a great variation across experimental replications or too small variation between parental lines with respect to this trait (Bolibok and Rakoczy-Trojanowska 2006). Similarly, no QTL for regeneration was found in the study on rice seed callus culture performed by Taguchi-Shiobara et al. (2006). Interestingly, this result is new in comparison with data from other reports. For example, Agache et al. (1989) revealed that genes located on 1RS chromosome arm are involved in the regeneration ability. Zhang et al. (2003) found two QTLs significantly associated with the aptitude for green plant regeneration on wheat chromosome arm 5BL, subsequent studies localized it on chromosomes 2A, 2B and 5B (Anca et al. 2007), whereas in triticale González et al. (2005) detected four regions on chromosomes 1B, 1R, 4R and 7R significantly associated with this trait.

According to our research, the triticale chromosome 7R seems to be connected with induction as well as with final efficiency of androgenesis. Bolibok et al. (2007) found on this chromosome locus influencing callus induction in the culture of immature inflorescences. The obtained results stayed in agreement with González et al. (2005) reporting a strong correlation for androgenic embryo induction and total yield of the process; the proximity of these QTLs implies that they are jointly inherited and may be either due to tight linkage or due to pleiotropy.

In conclusion, the presented results made the next step in widening the knowledge of molecular background of androgenesis. From practical point of view, genotypes characterized by high effectiveness of androgenic responsiveness have been identified in ‘Saka 3006’ × ‘Modus’ DH population. These genotypes can be directly used as a donor source of valuable alleles in triticale breeding programs. Markers identified as linked with QTLs controlling effectiveness of androgenesis can facilitate transfer of positive alleles to other triticale genotypes with the use of marker-assisted selection (MAS) method. They can be also applied for evaluation of other genotypes in breeding programs. Nonetheless, in some cases our results need further confirmation, as some detected QTLs were expressed only in a particular environment/experiment, or were expressed differently in different replications, whereas QTL consistency across different environments and backgrounds is an important prerequisite for application of MAS in breeding programs.
